# Ca^2+^ Signaling in Striated Muscle Cells During Intracellular Acidosis

**DOI:** 10.3390/biom15091244

**Published:** 2025-08-28

**Authors:** Florentina Pluteanu, Boris Musset, Andreas Rinne

**Affiliations:** 1 Department of Anatomy, Animal Physiology and Biophysics, Faculty of Biology, University of Bucharest, 050095 Bucharest, Romania; florentina.pluteanu@bio.unibuc.ro; 2 Center of Physiology, Pathophysiology and Biophysics, Paracelsus Medical University, 90419 Nuremberg, Germany; boris.musset@pmu.ac.at; 3 Department of Biophysics and Cellular Biotechnology, University of Medicine and Pharmacy “Carol Davila” Bucharest, 050474 Bucharest, Romania

**Keywords:** acidosis, calcium signaling, contractility, cardiac muscle, skeletal muscle, excitation–contraction coupling (ECC)

## Abstract

The cytosolic pH (pH_i_) of mammalian cells is tightly maintained at values ~7.2. Cytoplasmic acidosis (pH_i_ < 6.8) occurs when the intracellular proton concentration ([H^+^]_i_) exceeds the buffering capacity of the cytosol and transport processes to extrude protons are exhausted. During intracellular acidosis, the contractility of cardiac and skeletal muscle cells is strongly reduced, often at sufficient Ca^2+^ levels. A contraction of striated muscle is achieved when the intracellular calcium (Ca^2+^) concentration rises above resting levels. The amplitude and kinetics of Ca^2+^ signals are controlled by Ca^2+^ handling proteins and force is generated if Ca^2+^ ions interact with contractile filaments of the sarcomere. Some aspects of this phenomenon, such as the biochemical origin of excessive protons in working muscle cells and molecular interactions of protons with Ca^2+^ handling proteins or contractile filaments, are not yet fully understood. This review summarizes our current understanding of how striated muscle cells handle Ca^2+^ and H^+^ and how a rise in [H^+^]_i_ may interfere with Ca^2+^ signaling in the working skeletal muscle (fatigue) or during ischemic events in cardiac muscle. Finally, we briefly address experimental strategies to measure Ca^2+^ signaling at different pH values with fluorescent probes and highlight their limitations.

## 1. Introduction

The concentration of free protons ([H^+^]) in aqueous solutions is low and commonly expressed on a logarithmic scale as pH = −log [H^+^] [1,2]. In mammalian cells, extracellular pH (pH_o_) is rigorously maintained between 7.3–7.4 [3,4,5], whereas intracellular pH (pH_i_) ranges between 7.0–7.2 (skeletal muscle cells and cardiac myocytes) [6,7]. When transient metabolic changes in free [H^+^] occur, pH_i_ is kept constant by means of buffering. In biological solutions, weak acids or bases, such as phosphate groups and side chains of amino acids of proteins, serve as proton buffers. In addition, cells employ transport systems for acids and bases, such as proton pumps and transporters or bicarbonate shuttles to fine-tune pH_i_ on the long-term range [4]. There is a modulatory role of pH_i_ on Ca^2+^ signaling in striated muscle cells [8,9,10,11] and non-excitable cells [12]. Cells use Ca^2+^ ions as secondary messengers to regulate different cellular functions, including contraction of smooth and striated muscle [13,14,15]. Ca^2+^ signaling occurs when the low, resting Ca^2+^ concentration (~100 nM) suddenly increases to values up to 1–2 µM and Ca^2+^ sensor proteins integrate the information encoded by Ca^2+^ signals, such as amplitude, frequency and duration, with cellular functions. Endogenous Ca^2+^ sensing proteins relevant for striated muscle cell function are calmodulin (CaM), which controls enzymes and ion channels [16] and troponin, which controls muscle contraction (see below) [17,18]. The intracellular Ca^2+^ concentration ([Ca^2+^]_CYT_) increases due to activity of plasmalemmal ion channels mediating Ca^2+^ influx and/or intracellular Ca^2+^ release channels, which liberate Ca^2+^ from intracellular stores, such as the endoplasmic reticulum (ER) or the sarcoplasmic reticulum (SR) in muscle cells. Ca^2+^ signals that control muscle contraction are brief and reversible (“Ca^2+^ transient”) and resting Ca^2+^ levels are established via removal of Ca^2+^ ions from the cytosol by Ca^2+^ pumps, such as the plasma membrane Ca^2+^ ATPase (PMCA, Ca^2+^ extrusion), the sarcoplasmic/endoplasmic Ca^2+^ ATPase (SERCA, store-refilling) and plasmalemmal Ca^2+^ exchangers [15,19,20,21]. In addition to Ca^2+^ release and removal mechanisms, intracellular Ca^2+^ signals are shaped by reversible binding of Ca^2+^ ions to intracellular proteins serving as Ca^2+^ buffers [22]. Ca^2+^ signaling is affected by pH_i_ via two mechanisms: First, H^+^ and Ca^2+^ compete for identical binding sites at protein buffers. This has the consequence that protonation of a buffer will increase its K_d_ for Ca^2+^ [23] and a rise in [H^+^] (a fall in pH) will unavoidably increase the free [Ca^2+^] and vice versa [3]. Second, the protonation of Ca^2+^ handling proteins at secondary sites [24] may allosterically alter their function to transport Ca^2+^ [25].

Acidosis is the fall of pH below physiological values [26] and is recognized as a clinical parameter when plasma pH is <7.35 (acidemia) due to metabolic or respiratory disturbances [27]. Respiratory acidosis can cause a rapid fall in pH_o_ due to an extracellular buildup of CO_2_. Because CO_2_ is highly diffusible across lipid bilayers, a rise in extracellular CO_2_ causes an increase of the cell’s acid load, which lowers pH_i_ [4,28]. Intracellular acidosis is defined as a fall of pH_i_ < 6.8 and it occurs when there are more free cytosolic protons than buffers can bind and transport processes can remove from the cytoplasm [4]. Acidosis is frequently observed under physiological conditions due to metabolic processes in working muscle or pathologically in ischemic tissue under hypoxic conditions or during inflammation. It is worth noting that any fall in pH_o_ during extracellular acidosis will directly cause an accompanying fall in pH_i_ since protons can easily cross the plasma membrane via diffusion or transport processes [3]. Therefore, a direct relation between low pH_o_ values and intracellular acidosis has been demonstrated for cardiac, skeletal and smooth muscle cells [29,30]. This review will focus on the effects of intracellular acidosis on striated muscle Ca^2+^ handling and contraction. Intracellular acidosis causes a remarkable reduction in striated muscle contractility, which is the ability of the muscle to generate force. As part of this special issue, our review aims to introduce the Ca^2+^ signaling events that lead to contraction in striated muscle cells and to provide an overview of the most important cellular mechanisms that contribute to the reduction in contractility during acidosis in skeletal and cardiac cells. We will also briefly address optical techniques to assess Ca^2+^ signaling and pH_i_ with fluorescent probes in living cells to study those processes.

## 2. Excitation–Contraction Coupling (ECC) in Striated Muscle Cells

**Figure 1:**
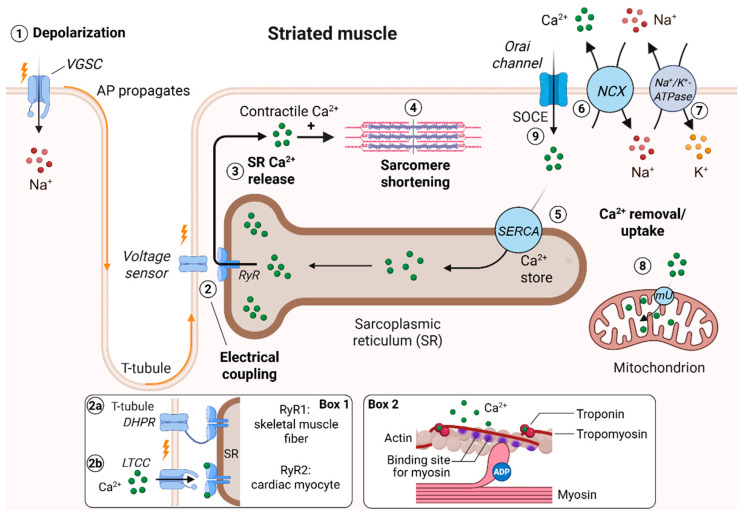
Excitation–contraction coupling in striated muscle cells. (1) Membrane depolarization generates an action potential (AP) that propagates into T-tubules. (2) Depolarization is sensed by voltage-sensing proteins DHPR (skeletal muscle cells, box 1, 2a) or LTCC (cardiac cells, box 1, 2b). (3) This opens Ca-release channels (RyRs) at the sarcoplasmic reticulum, which causes a large elevation of [Ca^2+^]_CYT_. (4) Ca^2+^ release activates the contractile proteins of the sarcomere to initiate muscle contraction. Box 2 shows a snapshot of the cross-bridge cycle, the Ca^2+^-dependent interaction of actin and myosin. The muscle cell relaxes when resting Ca^2+^ levels are restored. (5) This is mediated by transporting Ca^2+^ ions into the SR by SERCA and (6) by transporting Ca^2+^ out of the cell via NCX. (7) NCX uses the Na^+^ gradient across the plasma membrane for its transport, which is provided by the Na^+^/K^+^-ATPase. (8) Ca^2+^-uptake into mitochondria may buffer elevations in [Ca^2+^]_CYT_ in cardiac myocytes. (9) Ca^2+^ influx via SOCE supports refilling of the SR by SERCA in skeletal muscle cells. DHPR: dihydropyridine receptor. LTCC: L-type Ca^2+^ channel. mU: mitochondrial calcium uniporter. NCX: Na^+^/Ca^2+^-exchanger. SERCA: sarcoplasmic/endoplasmic Ca^2+^ ATPase. SOCE: store-operated Ca^2+^ entry. VGSC: voltage-gated sodium channel.

**Figure 2:**
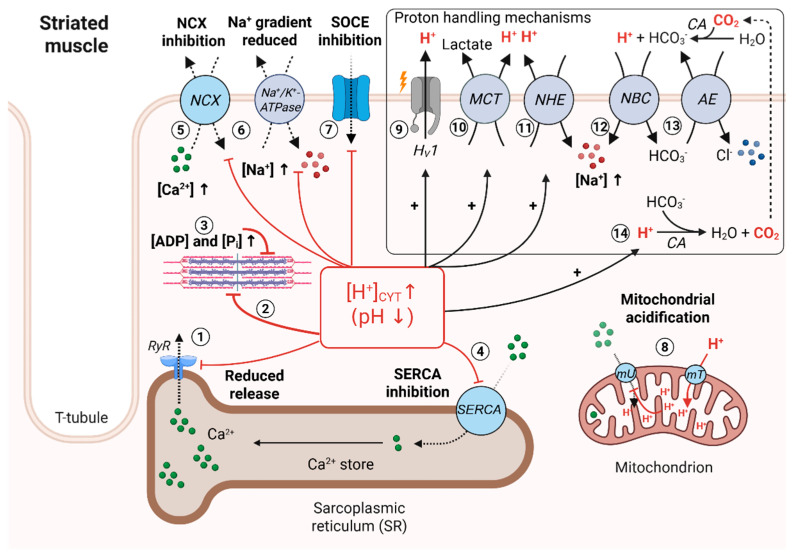
Effects of intracellular protons on Ca^2+^ release and contractility in striated muscle cells. An increase in [H^+^]_CYT_ has multiple effects on Ca^2+^ handling proteins: (1) Inhibition of RyRs reduces the amount of Ca^2+^ to be released from the SR. (2) Protonation of troponin C reduces its affinity for Ca^2+^. In addition, protonation of myosin slows down ATP hydrolysis at myosin heads and, thus, performance of the cross-bridge cycle. (3) Accumulation of inorganic phosphate P_i_ and ADP at myosin heads impairs repetitive cross-bridge formation, an effect that is additive to protonation of myosin. (4) Inhibition of SERCA reduces SR Ca^2+^ content. (5) Inhibition of NCX causes accumulation of Ca^2+^ in the cytosol (cardiac cells). (6) Protons inhibit the Na^+^/K^+^-ATPase, which causes intracellular Na^+^ accumulation and reduces the gradient for Na^+^ across the plasma membrane. This may further limit the transport rate of NCX in direct mode. (7) Protonation inhibits SOCE, which impairs refilling of the SR in skeletal muscle cells. (8) Protons can enter mitochondria via mitochondrial transporters (mT). Acidification of the mitochondrial matrix impairs mitochondrial Ca^2+^ uptake via the uniporter, which may contribute to the observed increase in [Ca^2+^]_CYT_ during acidosis (cardiac myocytes). 9 to 14: Cellular proton handling mechanisms: (9) Membrane depolarization opens the voltage-gated proton channel H_v_1 during the cardiac action potential. (10) Protons leave the cell via extrusion by the monocarboxylate transporter (MCT) in symport with lactate and by the sodium–proton exchanger (NHE, 11). Strong activation of NHE adds to the intracellular Na^+^ accumulation. 12 to 14: Long-term regulation of pH by the CO_2_/bicarbonate (HCO_3_^-^) buffer system. Bicarbonate can enter the cell via the sodium/bicarbonate cotransporter (NBC, 12) and can be excluded from the cell via Cl^−^/bicarbonate anion exchange (AE, 13). Additionally, bicarbonate is formed by intracellular carbonic anhydrases (CAs) that metabolize CO_2_ stemming from mitochondrial respiration (not shown in Figure 2). (14) CAs catalyze the reaction of protons with bicarbonate, which yields H_2_O and CO_2_. The latter leaves the cell by diffusion. Extracellular CA catalyzes the hydration of CO_2_, which provides bicarbonate for NBC and AE transporters and releases protons into the extracellular space.

The signaling process that mediates contraction of striated muscle cells is called excitation-contraction coupling (ECC) [31,32,33]. The term summarizes a chain of signaling events in which an electrical signal of the plasma membrane, an action potential (AP), is converted into mechanical contraction via intracellular Ca^2+^ release (Figure 1). APs propagate into the depth of muscle cells via invaginations of the plasma membrane named T-tubules (Figure 1, 1). In striated muscle cells, excitation of the T-tubule is coupled with a mechanism that releases Ca^2+^ from the SR (electrical coupling, Figure 1, 2). In skeletal muscle cells, electrical coupling is achieved by activation of the dihydropyridine receptor (DHPR), which is directly coupled to a Ca^2+^ release channels of the sarcoplasmic reticulum (SR), the ryanodine receptors type 1 (RyR1s, Figure 1, box 1, 2a) [34]. In cardiac cells, electrical coupling involves the L-type Ca^2+^ channel (LTCC), which mediates Ca^2+^ influx. Ca^2+^ ions diffuse to adjacent SR membranes and open the Ca^2+^-gated, cardiac ryanodine receptor type 2 (RyR2, Figure 1, box 1, 2b) [35,36]. Opening of either type of RyR causes rapid release of Ca^2+^ from the SR into the cytosol down its concentration gradient (Figure 1, 3), which is converted into mechanical contraction at the sarcomere (Figure 1, 4). The kinetics, amplitude and duration of this intracellular Ca^2+^ elevation define the speed, strength and duration of muscle contractions, respectively [37].

The molecular steps controlling Ca^2+^-dependent contractions are identical for cardiac and skeletal muscle cells: a rise in intracellular Ca^2+^ induces cross-bride formation between actin and myosin, a process that is regulated by the Ca^2+^-sensitive troponin–tropomyosin complex [38]. At low [Ca^2+^]_CYT_, troponin masks the myosin interaction sites on the actin molecule and cross-bridge formation is prevented. At high [Ca^2+^]_CYT_, Ca^2+^ binds to the regulatory protein troponin and induces a conformational change of the troponin–tropomyosin complex, which liberates myosin binding sites on the actin molecule (Figure 1, box 2) [39]. Cross-bridge formation will shorten sarcomeres, which are the contractile units of striated muscle cells (Figure 1, 4) [17,40,41]. The force a striated muscle can generate depends on the sum of all cross-bridges, a process with steep Ca^2+^ dependence [42,43]. The second important molecule that regulates the cross-bridge cycle is ATP, which binds to myosin [44]. The affinity of myosin molecules for actin is low when ATP is bound to the myosin head (loose cross bridges) and high if ATP is hydrolyzed and ADP is bound to myosin (Figure 1, box 2). The hydrolysis rate of ATP and the ATP-to-ADP exchange rate at myosin heads determine the speed of new cross-bridge formations (i.e., the speed of contraction) [45,46,47].

The muscle relaxes when [Ca^2+^]_CYT_ falls back to resting levels. This is controlled by Ca^2+^-dependent inactivation of RyRs at high cytosolic Ca^2+^ concentrations [35,36,48] and by activation of Ca^2+^ transport mechanisms that remove Ca^2+^ from the cytosol. The major transport processes in striated muscle cells involve SERCA at the SR (Figure 1, 5) and the plasmalemmal sodium calcium exchanger (NCX) [33,37,49] (Figure 1, 6). The latter uses the Na^+^ gradient across the plasma membrane for Ca^2+^ extrusion, which is generated by the Na^+^/K^+^-ATPase (Figure 1, 7). For skeletal muscle cells there is experimental evidence that refilling of the SR with Ca^2+^ ions is supported by Ca^2+^-influx via store-operated Ca^2+^ entry (SOCE, Figure 1, 9) [50]. In contrast, cardiac SOCE generates only locally restricted Ca^2+^ signals that do not seem to contribute to contractile Ca^2+^ signaling [51]. Established functional roles for cardiac SOCE are the regulation of pacemaker activity of the sinoatrial node [52] and activation of gene transcription processes associated with cardiac hypertrophy and heart failure [53,54].

Additionally, mitochondria can store Ca^2+^ ions and thus may affect [Ca^2+^]_CYT_ and ECC. Any elevation in cytoplasmic Ca^2+^ may cause an uptake of Ca^2+^ into mitochondria via Ca^2+^ diffusion through the mitochondrial uniporter (Figure 1, 8) [55]. The exact [Ca^2+^] within the mitochondrial matrix is cell type-specific. In resting striated muscle cells, it is equal or close to [Ca^2+^]_CYT_ [56]. In rat cardiac myocytes, expression of a mitochondria-specific Ca^2+^ biosensor revealed that resting mitochondrial Ca^2+^ levels were between 70 nM and 100 nM and the mitochondrial Ca^2+^ concentration increased with each cytosolic Ca^2+^ transient when ECC was evoked by electrical stimulation of the cells, an effect that was attributed to diffusion of Ca^2+^ from the cytosol into mitochondria [57]. The effect of mitochondrial Ca^2+^ uptake on [Ca^2+^]_CYT_ is more pronounced in cardiac cells, in which mitochondria occupy ~30% of the cell volume [58,59]. Whether mitochondrial Ca^2+^ buffering is relevant in skeletal muscle cells is a matter of debate [60]. One limiting factor might be that mitochondria occupy only a small volume (~2% to 6%) of skeletal muscle cells [61]. Functional roles of mitochondrial Ca^2+^ uptake/buffering include shaping of Ca^2+^ transients, fine-tuning of CICR at ER/mitochondrial contact sites [62] and providing a feedback signal that couples mitochondrial ATP production to its cytoplasmic demand during cell contractions [58,59].

Taking this complexity of ECC into consideration, there are many molecular steps that can be modified by acidosis. We will summarize next how a fall in pH_i_ below normal values can alter ECC, Ca^2+^ signaling and Ca^2+^ buffering. This is relevant for the contractile function of striated muscle, which is severely depressed by intracellular acidosis occurring in physiological and pathological conditions.

## 3. Mechanisms Inducing Intracellular Acidosis in Striated Muscle Cells

**Skeletal muscle.** Fatigue is the inability of the muscle to generate sustained force or power during repetitive contractions [63,64]. Within this period, the speed of individual muscle contractions slows down and maximal force is reduced. Fatigue is well pronounced in fast muscle fibers and less or not at all pronounced in slower muscle fibers [65]. Initially it was assumed that fatigue is the simple result of excessive consumption of energy (ATP) during heavy workloads of the muscle [66]. In this scenario, fatigue occurs whenever muscle fibers consume ATP faster than it can be supplied by metabolic processes. While this mechanism may contribute to fatigue in fast-twitching muscle fibers, some studies indicated that intracellular ATP levels are not likely to fall to a level that may compromise contraction in most muscle types [67] and muscle fibers displayed fatigue at constant ATP levels [68]. Instead, there is evidence that skeletal muscle fatigue is the result of an accumulation of protons and P_i_ in the working muscle. Acidosis has been associated with fatigue and occurs in a reversible manner during intense exercise [69], in which pH_i_ can fall from ~7.0 (at rest) to ~6.4 (fatigued) in human skeletal muscle [7]. During longer periods of exercise, ATP synthesis depends on glycolysis that generates pyruvate, which either fuels ATP generation in mitochondria (via the tricarboxylic acid cycle) or which is converted to lactate by the enzyme lactate dehydrogenase (LDH) in the cytosol when mitochondrial uptake is saturated [70]. Since it was discovered that the lactate concentration rises in working skeletal muscles [71], the term “lactic acidosis” has been associated with fatigue. The underlying concept implies that accumulation of lactic acid or lactate due to increased glycolysis rates will cause intracellular acidification [7,72]. Others have argued that the biochemistry of skeletal muscle cells would not be compatible with this concept. Some counter arguments include the following: (1) The final product of glycolysis in the cytosol is pyruvate. Mitochondria import pyruvate and consume protons to supply ATP in working muscle, which limits pyruvate accumulation in the cytosol [73,74]. Furthermore, when mitochondrial import is exhausted, excessive pyruvate in the cytosol is converted by LDH to lactate, but not to lactic acid (which could lower pH_i_). Lactate, in turn, stimulates monocarboxylate transporters (MCTs), which are symporters that transport lactate and protons out of the cell (Figure 2) [75]. Due to this transport mechanism, the plasma concentration of lactate can rise about five times to ~8 mM during intense exercise [76]. Extracellular lactate can enter neighboring muscle cells or cells of other tissues via MCTs, which operate bidirectionally, where it fuels ATP synthesis following its conversion back into pyruvate [77]. The conclusion is that mitochondrial metabolism and MCT-mediated transport will limit the accumulation of lactate and protons, so that the fall in pH_i_ cannot be caused by glycolysis alone. (2) Based on experiments using skinned skeletal muscle fibers, there is no direct evidence that lactate impairs contractility, even at high concentrations [70]. It has been suggested that the high rates of ATP hydrolysis at myosin molecules during heavy exercise would generate excessive protons in the cytoplasm [70,78,79,80]. Irrespective of the biochemical origin of those protons, significant acidification of skeletal muscle cells occurs during long periods of intense exercise and intramuscular pH_i_ was reported to fall to values ≤ 6.4 in fatigued skeletal muscle [7]. Considering all this, mechanisms inducing intracellular acidosis are not restricted to ATP deficiency, lactate accumulation or high glycolysis rates, but also caused by direct accumulation of protons stemming from alternative biochemical processes. It is likely that mechanisms other than ATP hydrolysis may contribute to proton accumulation, such as release of protons from buffers or using protons as counter ions for Ca^2+^ transportation into organelles. Those mechanisms are not well investigated yet (discussed in Section 5), and they may contribute to local rather than global changes in pH.

**Cardiac muscle**. Intracellular acidosis does not occur during normal activity of the heart. Metabolic changes that occur during physiological heartbeats do not cause acidification [81], probably due to the large buffering capacity of cardiac myocytes for protons (20–90 mM/pH unit) [31]. However, severe intracellular acidosis occurs in cardiac cells during pathologies that alter the acid–base homeostasis and lead to extracellular acidification, due to respiratory or metabolic disturbances, sleep apnea and ischemia. Respiratory acidosis is characterized by a buildup of extracellular CO_2_, which induces a rapid fall in pH_i_ [4]. This is caused via diffusion of CO_2_ across the plasma membrane and/or via cellular uptake of protons from the extracellular space. The latter is evenly effective, because the plasma membrane has a large conductance for protons [82] and the electrochemical driving force for protons favors proton influx [4]. Consequently, there is a direct relationship between the fall of pH_o_ and a corresponding fall of pH_i_ in muscle cells [30,83]. Cardiac ischemia lowers pH_i_ significantly: in diabetic cardiac myocytes, pH_i_ values as low as 6.0 have been observed during ischemic episodes, an effect that was reversible once normal oxygenation was established [84]. When pH_i_ falls this much, the cardiac contractility is markedly reduced [85]. Thus, in contrast to skeletal muscle, the mechanisms leading to intracellular acidification of cardiac myocytes are rather caused by extracellular pH changes than by intrinsic metabolic changes in pH_i_.

## 4. Effect of Low pH_i_ on Striated Muscle Contractility

**Skeletal muscle.** High-intensity exercise affects ECC via mechanisms that are pH-independent and pH-dependent. In intact muscle tissue, there is an accumulation of extracellular K^+^ within T-tubules due to K^+^ efflux during APs, which depolarizes the tubules. ECC and Ca^2+^ release was shown to be efficient up to [K^+^]_o_~10 mM but strongly impaired when [K^+^]_o_ reached concentrations of 14–15 mM [86]. Such large K^+^ concentrations were observed locally, within the small volume of T-Tubules [87]. The resulting membrane depolarization limits the spread of excitation within the T-tubule and delays the recovery of the DHPR voltage sensor from inactivation, both of which render electrical coupling less efficient [88]. Intracellular acidosis has several consequences on ECC. (1) Inhibition of RyR1 by protons reduces Ca^2+^ release (Figure 2, 1). Single channel recordings demonstrated maximal RyR1 channel open probabilities *Po* at pH = 7.4, but almost complete closure of the RYR1 at pH = 6.5 [36]. This limits the amount of Ca^2+^ that is available for contractions and can reduce the peak force per individual twitch by more than 30% in skinned skeletal muscle fibers [89]. (2) There are multiple effects on proteins of the contractile apparatus (Figure 2, 2): first, protonation reduces the Ca^2+^-sensitivity of purified troponin C [90] and approximately three-times more Ca^2+^ is required to form all cross-bridges required for 50% of force generation (pCa_50_) in skinned muscle fibers [91]. Furthermore, the interaction of troponin C and troponin I, which defines the speed of cross-bridge formation, is less efficient, as shown in myofibril preparations [92]. Protonation of myosin reduces its affinity for actin, which affects the total number of cross-bridges and reduces the muscle tension by about 20% and 45%, depending on the type of skinned muscle fiber (fast or slow twitch) studied [93,94]. Importantly, those negative inotropic effects persisted even at saturating Ca^2+^ concentrations [95], suggesting that pH-related modifications of contractile proteins dominate the negative inotropic effect. (3) High rates of ATP hydrolysis in the working muscle result in accumulation of ADP, P_i_ and H^+^ within the sarcomere (Figure 2, 3). Protons and P_i_ act synergistically and have a large negative effect on contractility: P_i_ reaches peak concentrations between 15 mM and 30 mM in working skeletal muscle [96]. Raising P_i_ to 30 mM induced a large shift in the force–pCa relationship to the right for both slow- and fast-type, skinned muscle fibers [97], which means that more Ca^2+^ is required to generate force. This suggests that, like protons, P_i_ alone can strongly depress the generation of force. Furthermore, an accumulation of P_i_ stabilizes the ADP + P_i_-bound conformation of myosin, which slows down the transition from ADP-to-ATP-bound form. This reduces the velocity of cross-bridge formation and impairs the formation of new cross-bridges. In addition, it reduces the force that previously formed cross-bridges had already developed [97,98]. Moreover, P_i_ can react with protons to its diprotonated form, which seems to have a larger negative inotropic effect than P_i_ or protons alone when applied to skinned fibers [99]. In combination, all those molecular events reduce the efficiency of Ca^2+^ ions to activate the cross-bridge cycle and/or reduce the force that existing cross-bridges can produce. (4) A rise in intracellular protons reduces the pump function of SERCA (Figure 2, 4). When SERCA function was measured in vesicles obtained from skeletal muscle SR preparations, a change in pH from 7.0 to 6.0 reduced maximal pump rates (V_max_) by ~50%, an effect that was accompanied by a reduction in the pump’s Ca^2+^-affinity [100]. This is consistent with the observed decline in SR Ca^2+^-content and Ca^2+^ transient amplitude during acidosis in skeletal muscle fibers [101,102]. (5) In sarcolemmal vesicle preparations, the transport rate of NCX, which is stimulated by intracellular Ca^2+^, depends on pH_i_: transport by NCX was stimulated at pH = 9, but inhibited at pH = 6 [103]. This inhibition occurred allosterically following protonation of two histidine residues (H124, H165) within the transporter molecule, which are not located within the Ca^2+^ binding domains [104]. Any reduction in NCX transport rate may cause an elevation of [Ca^2+^]_CYT_ (Figure 2, 5). Because the cardiac isoform NCX1 has higher maximal Ca^2+^ transport rates than the skeletal isoform NCX3 [33,105], its inhibition by protons may cause a larger rise in [Ca^2+^]_CYT_ in cardiac cells than in skeletal muscle cells [106,107]. (6) Ca^2+^ currents underlying SOCE (Figure 2, 7) were dependent on pH_i_. Ca^2+^ release-activated Ca^2+^ currents (I_CRAC_) recorded at pH_i_ = 6.3 were reduced by more than 50% as compared to control currents recorded at pH_i_ = 7.3 [108]. Furthermore, I_CRAC_ was strongly dependent on pH_o_, which falls during ischemia or tissue inflammation. A reduction in pH_o_ from 7.4 to 6.3 almost completely abolished I_CRAC_. The dependencies of SOCE currents on internal and external pH have been attributed to protonation of several key residues distributed throughout the Orai molecule [109]. SOCE activity contributes to SR Ca^2+^ load in intact skeletal muscle fibers [50], suggesting that any reduction in SOCE-mediated Ca^2+^ influx may reduce the filling status of the SR in skeletal muscle cells during acidosis.

The mechanisms described above collectively contribute to exercise-induced or physiological fatigue at low pH_i_ values in skeletal muscle. It is important to note that contractility experiments are often conducted using single muscle fibers (intact or skinned) to precisely control experimental parameters. Can one extrapolate those results to the situation of intact muscles in vivo? Performance measurements in muscles from healthy subjects showed that exercise-induced, intracellular acidosis (pH_i_~6.5) reduced the total force, speed of contraction and peak power [110] to similar degrees than reported for single fibers. However, a comparison between different muscle types proves difficult because the degree of fatigue appears to be fiber type-specific: whereas intracellular acidosis exerted a pronounced negative inotropic effect in fast twitching fibers, it had little effect on slow twitching muscle fibers [110]. Therefore, not every type of muscle may display the same sensitivity to intracellular pH changes. Moreover, in intact muscle multiple metabolic changes occur at the same time: for example, mitochondria produce large amounts of reactive oxygen species (ROS) [111] and the increasing temperature in working muscle also seems to impair muscle performance [112]. ROS are known to modify the function of Ca^2+^ handling proteins, which impairs cellular Ca^2+^ handling [113,114]. The negative effect of ROS on skeletal muscle function is even more evident when the native ability of the cells to buffer ROS is reduced, as in metabolic disorders or during inflammation [115]. Thus, it may be difficult to estimate the relative contribution of each metabolite to fatigue in vivo (see Discussion below). The development of novel animal models for exercise-induced fatigue may help to better understand long-term molecular changes underlying skeletal muscle fatigue on systemic and organ levels in future studies [116]. If pathological acidemia occurs, the contractility of skeletal muscle can be further impaired by additional long-term structural and functional changes in the muscle that involve degradation of proteins and impaired energy supply due to mitochondrial dysfunction [117].

**Cardiac muscle.** Many of those effects described above apply to cardiac myocytes as well. There are, however, some notable differences: At normal pH_o_, there is no net accumulation of K^+^ in T-tubules during contractions with high frequencies, because K^+^ removal via Na^+^/K^+^-ATPase pump activity is more efficient in cardiac myocytes than in skeletal muscle cells [88]. Unlike in the working skeletal muscle, in which glycolysis is the main source for ATP, the majority (>90%) of cardiac ATP stems from aerobic processes [118] and the main source for intracellular acidification is CO_2_ released by mitochondria. CO_2_ can leave the cell by diffusion across the membrane, or it reacts with H_2_O to bicarbonate and protons, a reaction that is catalyzed by CAs [119]. It is assumed that under physiological conditions, the rate of protons generated by hydration of CO_2_ is in balance with transport rates of proton export mechanisms [120] (see below). However, significant intracellular acidosis causes a strong depression in the contractility of cardiac Purkinje fibers [121]. It is frequently observed during cardiac ischemia, which is characterized by a fall in pH_o_ and pH_i_, a reduced rate of ATP synthesis, negative inotropy [122,123,124] and, surprisingly, by a rise in [Ca^2+^]_CYT_ [123]. Intracellular acidosis has the following effects on cardiac ECC: (1) in isolated ventricular myocytes, protons affected the gating mechanism of L-type currents, and the amplitude of currents were 2.5 times smaller at pH_i_ = 6.2 as compared to pH_i_ = 7.2 [125], which reduces the efficiency of electrical coupling (Figure 1, box 1, 2b) [126]. In addition, low pH_i_ prevents PKA-mediated phosphorylation of LTCCs following β-adrenergic stimulation, which normally yields to larger Ca^2+^ currents and a gain in ECC efficiency [127]. The same mechanism blunts the compensatory positive inotropic effect that sympathetic nerve stimulation normally has on the human heart once the pump function turns weaker [128]. (2) Lipid bilayer experiments revealed an inhibition of RyR2s by cytoplasmic protons [129]. In intact and skinned cardiac myocyte preparations, the same effect caused less Ca^2+^ release from the SR per AP (Figure 2, 1), reflected by systolic Ca^2+^ transients with amplitudes that can drop by 50% at the beginning of acidosis [130,131,132]. However, during prolonged periods of acidosis the Ca^2+^ transient amplitudes recover [130], which is caused by an increase in diastolic [Ca^2+^]_CYT_ and subsequent increase in SR Ca^2+^ load [35,123]. (3) Experiments using either papillary muscle or cardiac Purkinje fibers revealed that the increase in diastolic [Ca^2+^]_CYT_ is partially caused by release of Ca^2+^ ions from internal buffers due to competition of H^+^ and Ca^2+^ ions for common binding sites (discussed in Section 5) [133,134] and by the following modifications of ion transport processes: the sodium–proton exchanger (NHE) is stimulated by intracellular protons (Figure 2, 11) [135] and NHE activation will lead to accumulation of internal Na^+^. Simultaneous inhibition of the Na^+^/K^+^-ATPase (Figure 2, 6), which would normally remove excessive Na^+^, further contributes to the accumulation of internal Na^+^ at low pH_i_ [136]. This reduces the Na^+^ gradient across the membrane that provides energy for other Na^+^-dependent transporters, as shown in inside-out patch-clamp experiments on ventricular myocytes [137]. Na^+^ accumulation affects Ca^2+^ extrusion by NCX: at low, physiological Na^+^ concentrations, allosteric inhibition of NCX by cytoplasmic Na^+^ fine-tunes the transport rate of NCX in forward mode and thus, regulates cardiac excitability and beat-to-beat Ca^2+^ changes in isolated myocytes and in intact hearts [138]. However, at higher internal Na^+^ concentrations, NCX will start to remove excessive Na^+^ at the cost of transporting Ca^2+^ into the cell (reverse mode of NCX, discussed below) [103]. In addition to its modulation by Na^+^, NCX itself is directly inhibited by protons as described above (Figure 2, 5) [104], an effect that is sensitized by intracellular Na^+^ when assessed in excised patches from ventricular sarcolemma [139]. In ventricular myocytes, the reduced transport rates of NCX in its forward mode caused a net elevation of diastolic [Ca^2+^]_CYT_, which stimulates SERCA and increases SR Ca^2+^ load by more than 100 µM [123]. The stimulatory effect of Ca^2+^ on SERCA seems to compensate for the three-times slower transport rates (V_max_) induced by protonation of cardiac SERCA alone, which was observed in experiments using SR vesicle preparations (Figure 2, 4) [100]. Following cardiac ischemia, the reverse mode of NCX causes severe damage of cardiac tissue. Due to the low pH_o_ during the ischemic period, the transport rate of NHE is reduced. Once reperfusion occurs, pH_o_ recovers quickly and NHE begins to rapidly transport protons out of the cell. This causes fast accumulation of Na^+^ (Figure 2, 11) and, in turn, rapid Ca^2+^ influx as NCX tries to remove the excessive Na^+^ in its reverse operation mode. In hearts from animal models and humans, this unusual large influx of Ca^2+^ via NCX represents a substrate for cardiac arrhythmias and causes more damage of the myocardium (reperfusion injury) [140,141]. A recent study suggests that inhibition of cardiac NCX1 by protons may contribute to Ca^2+^ overload and reperfusion injury in the same way as the Na^+^-driven regulation: during experimentally-induced acidosis (pH_i_ = 6.5), ventricular myocytes from genetically modified mice expressing a proton-insensitive mutant of NCX1 (H165A) did not show increases in Ca^2+^ transient amplitudes, suggesting that NCX was not inhibited and hearts from transgenic mice displayed reduced tissue damage following an ischemia–reperfusion protocol in comparison to wild-type mice [142]. This suggests, in turn, that the modulations of NCX by Na^+^ and H^+^ may induce Ca^2+^ overload in a synergistic fashion. (4) Like in skeletal muscle, in isolated papillary muscle the negative inotropic effect of intracellular acidosis is dominated by the reduced Ca^2+^ sensitivity of the cardiac contractile apparatus (pCa_50_ is shifted by +0.1 unit/per fall of 0.1 pH unit) [124]. In experiments using intact myocytes, contractility remained depressed during acidosis, although the systolic Ca^2+^-transient amplitudes increased above control levels [130]. (5) A rise in [H^+^]_CYT_ induces proton uptake into mitochondria via different transporter systems in exchange for Ca^2+^, Na^+^ or K^+^ ions (Figure 2, 8) [59]. Measurements using isolated mitochondria from ventricular myocytes showed that acidification of mitochondria reduces the electrochemical gradient that the mitochondrial uniporter uses for Ca^2+^ transport and limits mitochondrial Ca^2+^ uptake [143]. This effect may add to the elevation of [Ca^2+^]_CYT_ during acidosis. Interestingly, changes in pH_i_ in cardiac myocytes are not homogenous, as one would assume due to the high diffusion speed of protons in aqueous solutions [144], but may rather occur as pH gradients: intracellular pH measurements in isolated ventricular myocytes revealed that there is a subcellular heterogeneity of pH due to local interaction of protons with intracellular buffers, which slows down the diffusion speed of protons [145,146,147,148]. Consequently, pH gradients may locally enhance those inhibitory effects that protons have on Ca^2+^ handling proteins. If systemic acidemia cannot be treated, e.g., during chronic sepsis or kidney failure, then the pump function of the heart is impaired via two mechanisms. First, metabolic acidosis causes a fall in pH_o_, which reduces organ perfusion, causing tissue hypoxia [149]. Second, intracellular acidosis depresses the contractile function of cardiac myocytes by the mechanisms described above. On the level of the human heart, this translated into reduced left ventricular contractility and reduced cardiac output in patients with systemic acidemia (pH_o_ < 7.28) as compared to a cohort of patients with normal blood pH (pH_o_ > 7.28). Among the functional parameters that were affected by intracellular acidosis were stroke volume (reduced by ~40%) and ejection fraction (reduced by ~30%) [150]. Both parameters directly determine cardiac output. Thus, alterations of cardiac pump functions due to intracellular acidosis may contribute to the poor survival rate that is associated with chronic acidemia [151].

If intracellular acidosis is reversible, excessive protons are removed from the cytosol of ventricular myocytes by the following mechanisms: activation of the voltage-gated proton channel H_V_1 (Figure 2, 9; see Section 7) [152]; export via MCT (Figure 2, 10); Na^+^/H^+^-exchange by NHE (Figure 2, 11) and reaction with the cytosolic CO_2_/bicarbonate buffer system [153]. In the mammalian heart, intracellular bicarbonate is provided by Na^+^/bicarbonate cotransport (NBC, Figure 2, 12) [154] and by the CA reaction, which generates bicarbonate by hydration of CO_2_ stemming from mitochondrial respiration [119,155]. Bicarbonate leaves the cell by Cl^−^/bicarbonate anion exchange (AE, Figure 2, 13) [156]. In striated muscle, at low pH_i_ values, CAs catalyze the reverse reaction of protons with bicarbonate to H_2_O and CO_2_ that leaves the cell by diffusion (Figure 2, 14) [157]. CAs are not restricted to the cytoplasm and have been localized to different compartments, such as plasma membrane, mitochondria and the SR, where they regulate local pH [158]. Extracellular CAs in capillaries and erythrocytes reverse this reaction and hydrate CO_2_ to bicarbonate and protons. The interplay between intracellular and extracellular CAs is important to maintain the CO_2_ gradient and diffusion of CO_2_ across the plasma membrane [159]. The CO_2_/bicarbonate system contributes to ~20% of the total proton buffer capacity in skeletal muscle [160] and has a slightly larger buffer capacity in cardiac myocytes [161]. The importance of the CO_2_/bicarbonate buffering system for cardiac function was directly demonstrated by the observation that the contractility of isolated murine myocytes and whole-heart preparations declined over time in the absence of a bicarbonate buffer due to an accumulation of intracellular protons, an effect that reversed when the same preparation was bathed in a CO_2_/bicarbonate buffer and pH_i_ was normalized [156]. Despite contributing to buffering of protons, a recent study demonstrated that bicarbonate may emerge as an important signaling molecule that stimulates mitochondrial ATP production in isolated, contracting cardiac myocytes via a mechanism that is independent from mitochondrial Ca^2+^ [162]. Dysregulation of the CO_2_/bicarbonate buffer system or proton transport processes have been implicated in skeletal muscle or cardiac pathologies. For example, upregulation of CAs represents a biomarker for cardiac ischemia and heart failure in humans [163,164]. During cardiac hypertrophy, upregulation of NBCs to rectify pH_i_ caused Na^+^ accumulation and Ca^2+^ overload via NCX [165].

Chronic fatigue syndrome is characterized by a high sympathetic tone, to which skeletal muscles respond with secretion of vasodilators, including protons, to improve muscle perfusion (“sympathetic escape” [166]). One factor that contributes to this mechanism is an upregulation of NHE that may induce Na^+^ overload and Ca^2+^-overload, which further deteriorates the contractile function of an already weak muscle [167]. In line with this, the pharmacological inhibition of NHE prevented muscle degeneration in a mouse model of Duchenne’s muscle dystrophy [168]. Based on results from several animal studies, an inhibition of NHE1 has been suggested as a potential novel treatment option to reduce tissue hypoxia and cardiac damage during metabolic acidosis in humans [169]. Despite affecting Ca^2+^ handling proteins, a fall in pH_i_ will also alter the properties of Ca^2+^ buffers and Ca^2+^ storing organelles, which we will discuss below.

## 5. Effect of Low pH_i_ on Ca^2+^ Buffers and Ca^2+^-Storing Organelles

In most cells Ca^2+^ ions are bound to ~99% to intracellular buffers [23]. To give an example, calculations for cardiac myocytes suggest that a change in free cytosolic Ca^2+^ from resting levels of 100 nM to ~1 μM during systole will require the net mobilization of as much as 100 μM Ca^2+^ from internal stores in the presence of internal buffers [31]. The simplest interference between protons and Ca^2+^ ions in the cytoplasm is their competition for the same binding sites at intracellular buffers [25]. The most relevant cytosolic Ca^2+^ buffer in type II (fast twitch) skeletal muscle fibers is the protein parvalbumin [170]. Parvalbumin is considered a slow buffer because it binds both Ca^2+^ and Mg^2+^ thus its Ca^2+^ buffering properties depend on competition with Mg^2+^ [171,172]. Considering this, Ca^2+^ buffering by parvalbumin does not limit the rapid rising phase and amplitude of Ca^2+^ transients during single twitches, but affects the decay of the transient and thus, the relaxation time of skeletal muscle [173]. In contrast, there is no relevant role for parvalbumin in type I (slow twitch) muscle fibers due to their slow relaxation [170]. In heart tissue, parvalbumin expression was species-specific [174], with low expression levels in human and larger animals and higher expression in small animals, according to the relaxation speed [175]. The most relevant Ca^2+^ buffers in cardiac cells are the Ca^2+^ binding sites of troponin C [176] and SERCA [177]. Computer models suggest that simple binding (buffering) of Ca^2+^ ions to troponin C and SERCA, followed by active transport of Ca^2+^ into the SR shape Ca^2+^ transients in the decay phase of the Ca^2+^ signal [177]. Because protonation lowers the K_d_ of buffers for Ca^2+^, it is plausible that intracellular acidification will liberate a substantial amount of Ca^2+^ into the cytosol, which may affect [Ca^2+^]_CYT_ during acidosis [178]. The effect that acidosis lowers the Ca^2+^ buffer capacity of parvalbumin has been shown in dorsal root ganglia neurons [179]; however this effect has not been established for striated muscle. For cardiac troponin C protonation decreases its affinity for Ca^2+^ [180], which should result in liberation of Ca^2+^ from contractile filaments during acidosis and impaired contractility.

Mitochondria are organelles that can buffer transient changes in cytoplasmic Ca^2+^ via Ca^2+^ diffusion through the mitochondrial uniporter (Figure 1, 5) [181,182] and can release mitochondrial Ca^2+^ into the cytosol by Na^+^/Ca^2+^ exchange [181,183]. In cardiac muscle, it is a matter of debate whether mitochondrial Ca^2+^ uptake occurs fast on a beat-to-beat basis or in an integrative manner as slow increase over time, dependent on the frequency of contractions [184]. It has been suggested that mitochondrial Ca^2+^ uptake may contribute to feedback mechanisms that couple cytoplasmic ATP demand with mitochondrial ATP production by activating Ca^2+^-sensitive enzymes that control the tricarboxylic acid cycle [55,185,186]. At the same time mitochondria maintain the proton gradient across the inner mitochondrial membrane by pumping out the protons via complexes I, III and IV of the respiratory chain, to provide, in combination with the mitochondrial membrane potential, the energy for ATP synthesis [187]. There is some experimental evidence that cytosolic pH_i_ and mitochondrial Ca^2+^ signaling can interfere: acidification of the cytoplasm was accompanied by proton uptake via Na^+^/H^+^ exchanger, K^+^/H^+^-exchanger and Ca^2+^/H^+^ exchanger and proton leak [188,189,190], leading to acidification of mitochondria, which inhibited mitochondrial Ca^2+^ uptake by the mitochondrial uniporter in mitochondria isolated from cardiac cells [143] (Figure 2, 7). In isolated, electrically paced cardiac myocytes, oscillatory changes in pH_i_ (“pH transients”) displayed kinetics that resembled the beat-to-beat changes in cytosolic Ca^2+^ transients [191]. The authors suggested as an underlying mechanism that Ca^2+^ uptake by mitochondria will induce simultaneous release of protons into the cytosol, which caused the observed fluctuations in pH_i_. Those fluctuations were small (±0.1 pH units) and it remains to be established if they contribute to intracellular acidosis.

Lysosomes are traditionally seen as organelles involved in protein turnover and degradation of biomaterial (“waste removal”) [192,193] but not recognized as cellular compartments involved in Ca^2+^ signaling, even though they display high proton and Ca^2+^ contents [20,194]. Interference of protons with Ca^2+^ ions occurs during transport processes across the lysosomal membrane. Lysosomes maintain a low luminal pH ~4.5 to create a pH optimum for acid hydrolases [195]. In addition, they contain a considerably large amount of luminal Ca^2+^ (~500 μM) [194], which is partially released during vesicle fusion processes [196]. It has been suggested that lysosomes contribute to Ca^2+^ buffering in skeletal muscle cells and cardiac myocytes [197]. Lysosomal V-ATPases pump protons from the cytosol into the lumen of lysosomes [198]. This adds positive charges to an organelle with a very small lumen and to maintain the electrochemical gradient across the lysosomal membrane, any proton influx must be compensated either by import of anions, such as Cl^−^, or by export of cations, including Ca^2+^, via lysosomal ion channels and exchangers [199,200]. Even though the overall volume of lysosomes per cell volume is very small (~3%) [194], local release of lysosomal Ca^2+^ via type 2 two-pore channels has been implicated in triggering larger cytoplasmic Ca^2+^ release events from intracellular Ca^2+^ stores via CICR by modifying IP_3_-Rs in non-excitable cells [201] or cardiac RyR2 signaling in atrial and ventricular myocytes [202]. In skeletal muscle cells, lysosomes form contact sites with the SR, mitochondria and the nuclear membrane, but the effector proteins of lysosomal signaling at those sites are not well defined [203].

Another organelle that can contribute to transport of protons into the cytosol is the SR. The pump cycle of Ca^2+^-ATPases requires charge compensation: whenever SERCA pumps Ca^2+^ ions into the SR, the added charge is compensated by antiport of two protons to the cytosol for each Ca^2+^ ion that enters the SR lumen [204]. Whether acidification affects [Ca^2+^]_CYT_ and ECC by Ca^2+^ release from buffers or cell organelles in striated muscle cells has not been addressed yet.

This complexity of local and global variations of pH requires the use of appropriate methods that are sensitive and specific to measure Ca^2+^ and pH in different cellular compartments with sufficient temporal and spatial resolution. The next chapter will give a short overview of established and recently developed optical tools that can be used to measure Ca^2+^ and pH in living cells.

## 6. Optical Methods to Measure Ca^2+^_CYT_ and pH_i_ in Living Cells

To study how changes in pH will affect Ca^2+^ signaling, fluorescent indicators have been used to directly monitor changes in pH or [Ca^2+^] in living cells. Fluorescent Ca^2+^ indicators are available as chemical compounds (Ca^2+^ dyes) that are loaded into cells or as genetically encoded biosensors based on fluorescent proteins, which are expressed by the cells. For both types of indicators, intensity-based and ratiometric variants are available [205,206]. There are advantages for the choice of either type for experiments: chemical indicators are well-established [207], require less experimental intervention and offer a wide range of affinities for Ca^2+^ ions [208]. The major advantage of genetically encoded probes is the possibility to target them to cell organelles, which allows simultaneous Ca^2+^ measurements in the cytosol and in organelles, such as ER or mitochondria [209]. In general, sensors with high affinity for Ca^2+^ are used in the cytosol, whereas sensors with low affinity for Ca^2+^ can be used to measure Ca^2+^ in organelles with high Ca^2+^ content, such as the SR or lysosomes. Popular organelle-specific Ca^2+^ biosensors based on fluorescent proteins are the indicators of the GECO family [209,210,211,212]. During experiments at constant pH, changes in fluorescence of the Ca^2+^ indicator reflect changes in [Ca^2+^]. However, the assessment of [Ca^2+^]_CYT_ during dynamic changes of pH_i_ is challenging, because most Ca^2+^ indicators are pH-sensitive: for example, in situ calibrations demonstrated that the K_d_ for Ca^2+^ of the widely used indicator fura-2 was approximately two-times lower at pH = 7.2 than at pH = 6.6, which means that a considerably larger Ca^2+^ concentration is required to cause a similar change in the emission ratio of fura-2 [30]. Being proteins, genetically encoded Ca^2+^ sensors bind protons as well and the intensity of emitted fluorescence depends on pH, a phenomenon that is either caused by direct quenching of the fluorophore [213] and/or due to competition of protons with Ca^2+^ ions at Ca^2+^ binding domains of those sensors. Thus, careful pH-calibration of Ca^2+^ sensors may be required for fluorescence measurements at different pH_i_ values. On the other hand, the generation of fluorescence intensity/pH curves established specific p*K*a values for each type of fluorescent protein used in biosensors [214]. This allows one to design fluorescent biosensors for specific applications, such as Ca^2+^ sensors that are bright enough at the very low pH values in lysosomes [215]. Comprehensive reviews on chemical Ca^2+^ indicators and Ca^2+^ biosensors are given by Paredes and colleagues [208] and by Mank and Griesbeck [216], respectively.

There is also a wide range of chemical indicators or protein-based biosensors for the optical assessment of pH_i_ available. One simple way to measure cytoplasmic pH is the use of enhanced YFP (eYFP, pKa = 7.1), which is pH-sensitive and gets dimmer when protonated [217]. As for Ca^2+^ measurements, using targeted fluorescent proteins allows measurements of pH changes within cell organelles [217]. An example of a chemical dye to measure pH_i_ is the widely used compound BCECF (p*K*a~7.0), which is a dual-excitation, single emission ratiometric indicator suitable for intracellular pH changes from pH = 6.5–7.5 [218]. Newer pH-sensitive fluorescent proteins include red-shifted variants of fluorescent proteins or ratiometric sensors and are engineered with enhanced quantum yield, improved dynamic range and tailored to match the specific pH values of organelles, such as the ER, the Golgi apparatus, mitochondria or lysosomes. A recent overview on protein-based pH-biosensors is given by Li and colleagues [214] and chemical pH indicators are described in detail by Han and Burgess [218]. For some experiments it may be useful to monitor pH_i_ and [Ca^2+^]_CYT_ simultaneously. This can be achieved by using combinations of Ca^2+^ indicators and pH indicators with different spectral properties, such as the Ca^2+^ indicators fura-2 or indo-1 in combination with the pH indicator SNARF, which does not show a high affinity for Ca^2+^ ions [219,220]. The advantage of this approach is that one can distinguish the kinetics of Ca^2+^ changes from the kinetics of pH changes in real time. These tools are necessary to quantify the degree of acidosis during physiological activity and pathological states of striated muscle cells and to correlate pH changes with functional changes. Transgenic animal models expressing genetically encoded biosensors can be used for long-term studies of acidosis in striated muscle cells.

## 7. Discussion and Outlook

Intracellular acidosis is a strong inductor of negative inotropy in striated muscle cells. We have introduced general mechanisms on how protons may alter the function of Ca^2+^ handling proteins and the contractile apparatus. Many molecular aspects of this phenomenon are still controversially discussed for the two types of striated muscle cells. Studying the effects of protons on Ca^2+^ signaling can be challenging because properties of Ca^2+^ indicators are sensitive to pH, which requires careful interpretation of acquired data. Moreover, data on skeletal muscle contractility seem to be highly sensitive to experimental conditions: some results obtained from experiments using isolated muscle cells (skinned or intact fibers), such as the degree of negative inotropy that individual metabolites will induce, could not be confirmed in intact skeletal muscle preparations [221,222]. Storage of tissue samples for future experiments may also affect the contractile properties of muscle fibers: a recent paper demonstrated that storage in glycerol, a commonly used freezing agent, affected the passive stiffness of skeletal fibers [223]. Furthermore, it seems critical whether results were obtained at room temperature, which is common for fiber studies, or at body temperature, which is used for muscle preparations or studies in vivo [224]. This has led to some skepticism about how well single cell data may be extrapolated to the situation in intact muscle [221,222], thus further investigations will be required to address ischemia in the whole organ. During cardiac ischemia, excessive production of ROS affects cardiac excitability and adds to the negative inotropic effect as many Ca^2+^ handling proteins are redox-sensitive [113,114]. Experimentally, it may be difficult to separate proton-induced effects from ROS-mediated alterations of Ca^2+^ handling proteins in intact organ preparations.

An interesting outlook for future studies lies in alternative signaling pathways that cells can use to sense changes in pH: many cell types that experience sudden intracellular acidification can rectify intracellular acidosis by activation of proton channels [82]. Proton channel currents have been identified in skeletal myotubes [225] and adult skeletal muscle cells [226], but their physiological role is not well defined. The expression of proton channel mRNA in cardiac tissue has been described for multiple species [227], and there is some indication for upregulation of cardiac proton channel transcripts during heart failure [228]. Recently, functional currents of the voltage-gated proton channel H_V_1 were recorded in adult canine ventricular myocytes and pharmacological blockade of this channel in the absence of NHE activity lowered pH_i_ from 7.1 to 6.13 in beating myocytes [152]. Thus, H_V_1 contributes to the regulation of pH_i_ in ventricular myocytes when [H^+^]_CYT_ rises (Figure 2, 10), an alternative pathway to proton exchange by NHE [229,230]. The advantage of a voltage-gated proton channel, compared to a transporter like NHE, lies in its exclusive ability to conduct protons out of the cell, driven by the electrochemical gradient. In all primary mammalian cells measured to date, it does not permit proton influx [231]. Thus, during acidification, it requires no additional energy while counteracting the drop in cytosolic pH. This is particularly beneficial for excitable cells, as the channel expels protons from the cytosol during depolarization, which also serves as the gating mechanism for the channel. Depolarization typically occurs just before a rise in energy demand in muscle cells, when metabolically generated protons begin to accumulate. The mechanism underlying this unique biophysical property—functioning as a proton “overpressure valve”, exclusively conducting protons out of the cell—is still under investigation [232]. Nonetheless, voltage-gated proton channels have been identified in various mammalian cell types [233]. Another notable feature is the absolute selectivity for protons, with no other ions conducted [234]. A frequently overlooked capability of the voltage-gated proton channel is its ability to sense both intracellular and extracellular pH, effectively making it a cellular “pH meter.” In line with the topic of this review, it is worth noting that the first scientists to voltage-clamp this channel also investigated the relationship between intracellular calcium and pH in excitable cells [235].

Other membrane proteins that cells use to sense pH_o_ include Ca^2+^-conducting cation channels (ASIC- and TRP channels) [236], and the rather novel, but small class of proton-sensing, G-protein-coupled receptors (GPCRs) [237]. Some of those receptors couple to heterotrimeric G_q_ proteins and activate phospholipase C (PLC), which controls intracellular Ca^2+^ signals by activating IP_3_-mediated Ca^2+^ release from the SR [15]. The PLC-IP_3_-Ca^2+^ signaling axis controls gene transcription [238,239] and could potentially contribute to long-term adaptation processes of muscle cells to acidification. In skeletal muscle cells, the functional roles of conventional GPCRs or proton-sensing GPCRs are not well investigated, but there is evidence that some orphan receptors could belong to the class of proton-sensing GPCRs [240]. In the heart transcripts of GPR68, a proton-sensing GPCR which activates PLC, have been identified in neonatal ventricular myocytes [236] and adult tissue. Thus, proton-sensing GPCRs have been suggested as novel drug targets to treat ischemic heart diseases [241].

## 8. Conclusions

In conclusion, although the functional consequences of intracellular acidosis on the contractility of striated muscle cells are well established, the molecular aspects of proton biochemistry underlying acidosis remain unclear. There are interesting, unexplored signaling pathways that could link acidification during ischemia with Ca^2+^ signaling and gene transcription in striated muscle cells. The continuous development of fluorescent biosensors to assess Ca^2+^ and pH_i_ in living cells will prove useful to investigate those aspects in the future.

## Data Availability

The figures of this article were created in BioRender.com: Rinne, A. (2025); https://BioRender.com/itrywxc (accessed on 5 July 2025).

## Abbreviations

The following abbreviations are used in this manuscript:

ADP	Adenosine diphosphate
AE	(Cl^−^/bicarbonate) anion exchanger
ATP	Adenosine triphosphate
ASIC	Acid-sensing ion channel
BCECF	2″,7″-Bis-(2-carboxyethyl)-5-(and-6-) carboxyfluorescein
Ca^2+^	Calcium ion
[Ca^2+^]_CYT_	Intracellular (cytosolic) Ca^2+^ concentration
CO_2_	Carbon dioxide
CRAC	Ca^2+^ release-activated current
CICR	Ca^2+^-induced Ca^2+^ release
DHPR	Dihydropyridine receptor
ECC	Excitation-contraction coupling
ER	Endoplasmic reticulum
GECO	Genetically encoded calcium indicator for optical imaging
G_q_	G_q_ alpha subunit of heterotrimeric G proteins
GPCR	G-protein-coupled receptor
GPR68	G-protein-coupled receptor 68
H^+^	Proton
[H^+^]_CYT_	Intracellular (cytosolic) proton concentration
H_V_1	Hydrogen voltage-gated channel 1
IP_3_	Inositol trisphosphate
IP_3_R	IP_3_ receptor
K_d_	Dissociation constant
LTCC	L-type Ca^2+^ channel
MCT	Monocarboxylate transporter
mT	Mitochondrial transport
mU	Mitochondrial uniporter
nAChR	Nicotinic acetylcholine receptor
[NA^+^]_CYT_	Intracellular (cytosolic) sodium concentration
NBC	Sodium-bicarbonate cotransporter
NCX	Sodium-calcium exchanger
NHE	Sodium-proton exchanger
pCa_50_	Negative logarithm of the Ca^2+^ concentration at 50% force development
*P*_CO2_	Partial pressure of CO_2_
pH	Negative logarithm of the proton concentration [H^+^]
pH_i_	Intracellular (cytosolic) pH
pH_o_	Extracellular pH
P_i_	Inorganic phosphate
PIP_2_	Phosphatidylinositol bisphosphate
p*K*a	Negative logarithm of the acid dissociation constant Ka
PKA	Protein kinase A
PLC	Phospholipase C
ROS	Reactive oxygen species
RyR	Ryanodine receptor
SERCA	Sarcoplasmic/endoplasmic Ca^2+^ ATPase
SNARF	Seminaphtorhodafluor
SOCE	Store-operated Ca^2+^ entry
SR	Sarcoplasmic reticulum
TRP	Transient receptor potential
V-ATPase	Vacuolar-type ATPase
VGSC	Voltage-gated sodium channel
YFP	Yellow fluorescent protein
